# Supplementary material for: Pattern Learning Electronic Density of States
Source: Sci Rep. 2019 Apr 10;9:5879. doi: 10.1038/s41598-019-42277-9 (PMC6458116; doi:10.1038/s41598-019-42277-9)
Supplement: Supplementary file 1 — Supplemetary Information [file 41598_2019_42277_MOESM1_ESM.pdf]

Supplementary Information for

# Pattern Learning Electronic Density of States

Byung Chul Yeo,[1] Donghun Kim,[1] Chansoo Kim,[1] Sang Soo Han[1*]

[1]Computational Science Research Center, Korea Institute of Science and Technology (KIST), Seoul 02792, Republic of Korea

[*]Corresponding authors. E-mail: sangsoo@kist.re.kr

## 1. Details on features for representation of DOS patterns

In our learning model, we proposed three features relevant to the compositions and atomic structures to represent the DOS patterns. First, we introduced the $d$-orbital electron occupation ratio ($n_d$), defined by:

$$n_{d,A\ or\ B} = \frac{N_{d,A\ or\ B} \times x}{N_{d,A} \times x + N_{d,B} \times (1-x)}$$

where $N_{d,A}$ and $N_{d,B}$ are the numbers of $d$-orbital electrons of the A and B elements in the $A_xB_{1-x}$ alloy, respectively. For example, in the Cu-Ni alloy system, the electronic configuration of Cu is [Ar] $3d^{10}4s^1$, indicating that the number of $d$-orbital electrons of Cu is 10. Similarly, because the electron configuration of Ni is [Ar] $3d^84s^2$, the number of the $d$-orbital electrons of Ni is 8. Accordingly, for the $Cu_{0.5}Ni_{0.5}$ composition, $n_{d,Cu}$ and $n_{d,Ni}$ are 0.56 and 0.44, respectively.

To distinguish atomic structures, we introduced the coordination number (CN) feature, as shown in Fig. S1. The CN value was obtained by dividing the number of all bonds between the two atoms by the total number of atoms in the material system. Here, the bonds were calculated using the covalent atomic radii. In general, the CN of a simple cubic (sc) structure is 6, the CN of a body-centered cubic (bcc) structure is 8, and the CNs of a face-centered cubic (fcc) structure and a hexagonal-close packed (hcp) structure are equal to 12.

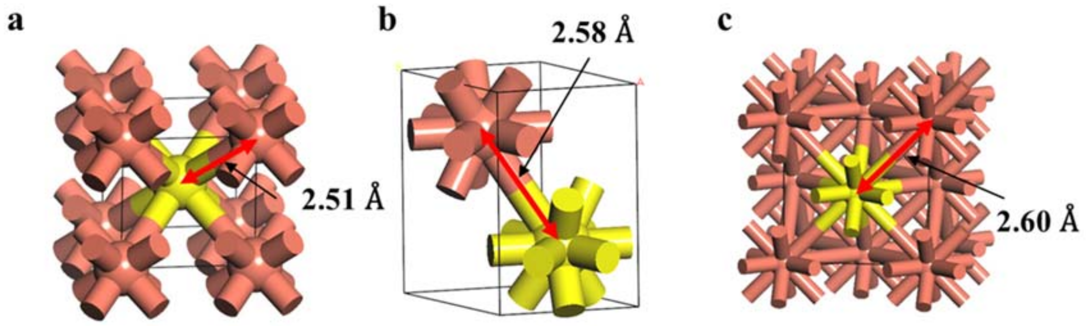


**Figure S1. Atom connectivity of various Cu crystal structures. a**, bcc**. b**, hcp. **c**, fcc. Bonds in each crystal structures are calculated with a covalent radius of Cu of 1.32 Å**.**

As another feature to distinguish atomic structures, we introduced the mixing factor ($F_{mix}$), which indicates the ratio of the number of different pair bonds (*A*-*B* bond) in the alloy system to the number of all bonds (*A*-*A*, *B*-*B*, and *A*-*B* bonds). As the miscibility of two elements in an alloy structure increases, $F_{mix}$ approaches 1. Conversely, as the immiscibility increases, $F_{mix}$ approaches to 0. Additionally, $F_{mix}$ of the pure metals is defined as 0. Using $F_{mix}$, one can readily distinguish two atomic structures even though they have the same CN value, as shown in Figure S2.

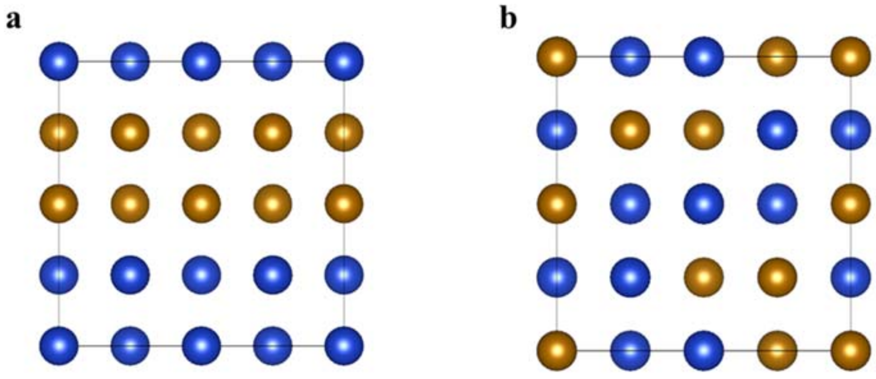


**Figure S2. Two different atomic structures of the A-B alloys but with the same CN value. a**, Immiscible structure with $F_{mix} = 0.33$. **b**, Randomly distributed structure with Fmix = 0.52. Since the **b** structure is more miscible, it has a higher $F_{mix}$.

## 2. Atomic structures of training and test systems

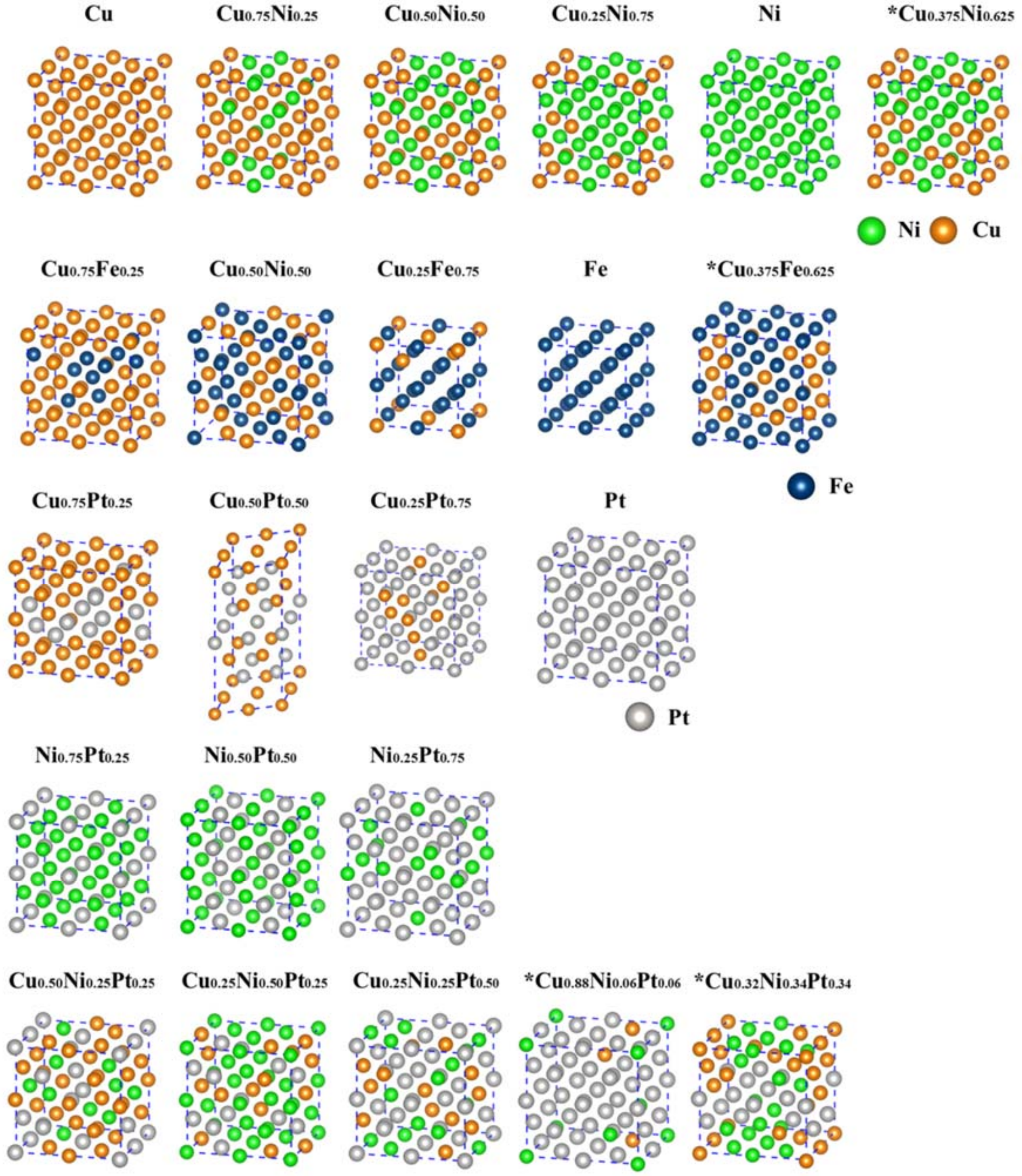


**Figure S3. Atomic structures of training and test data considered in bulk structures study. The asterisk (*) indicates test data. Otherwise, they are training data.**

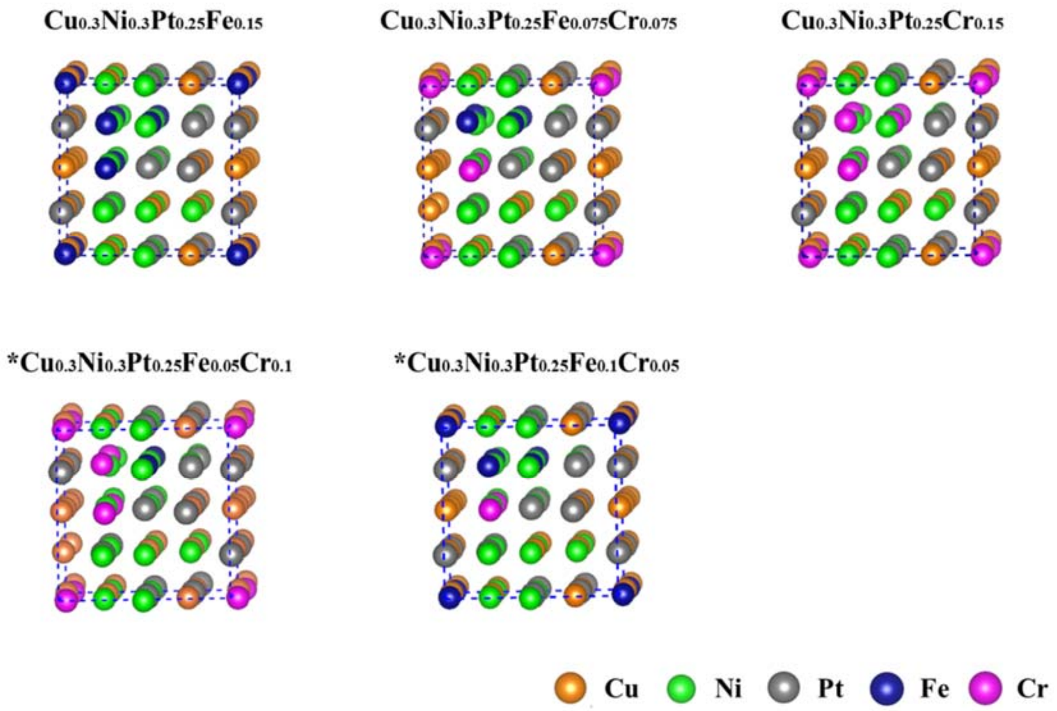


**Figure S4. Atomic structures of training and test data considered in high entropy alloy (HEA) structures study. The asterisk (*) indicates test data. Otherwise, they are training data.**

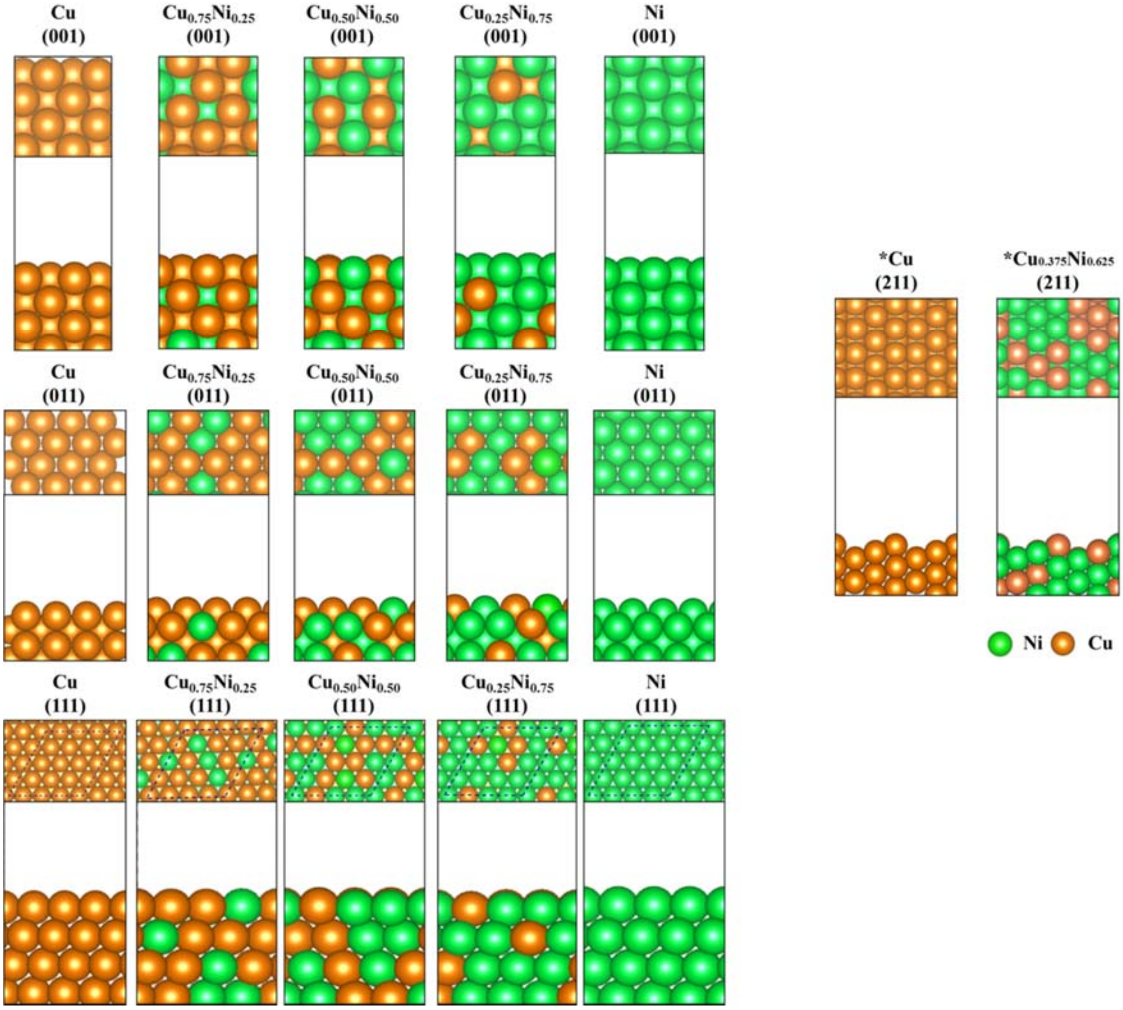


**Figure S5. Atomic structures of training and test data considered in slab structures study. The asterisk (*) indicates test data. Otherwise, they are training data.**

### 3. Details on DOS prediction using a probability matrix

Assuming that the maximum DOS value is T, $\rho_n(E_m)$ in the equation (4) can be expressed as $nT/N$. For example, if there are three non-zero values (0.3 for the 6th DOS level, 0.2 for the 4th level, and -0.1 for the 2nd level) at the given column vector in the DOS image matrix $\mathbf{I}'$ with a 100 × 100 grid, the non-zero probabilities for each DOS levels are 0.6 (=0.3/(0.3+0.2)) for the 6th level and 0.4 (=0.2/(0.3+0.2)) for the 4th level, where others with the exception of the positive entries in the $\mathbf{I}'$ are ignored. Then, assuming that T = 3, the DOS value at the energy interval is obtained as 0.156, according to the following calculation: 0.6 × (6×3/100) + 0.4 × (4×3/100).

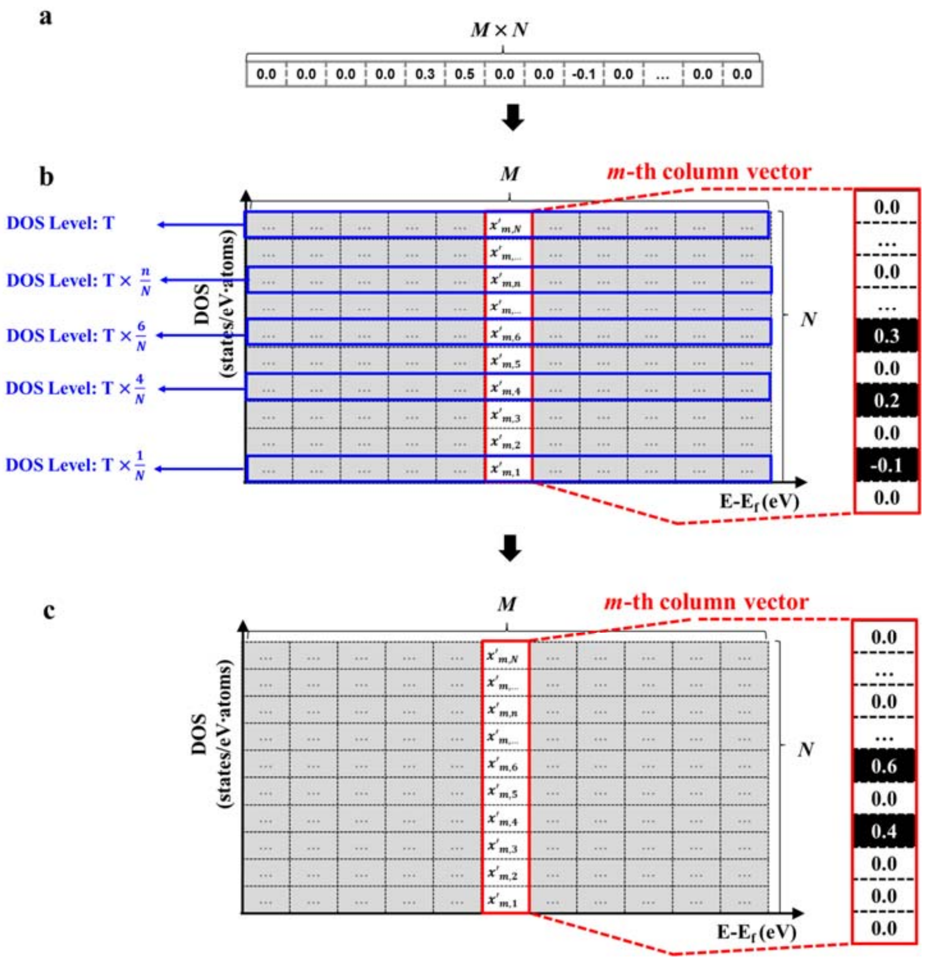


**Figure S6. Scheme for transformations from a DOS image vector ($\mathbf{x}'$) to a DOS image matrix ($\mathbf{X}'$) and from the DOS image matrix ($\mathbf{I}'$) to the DOS probability matrix ($\mathbf{X}'$). a,** the DOS image vector ($\mathbf{x}'$), **b,** the DOS image matrix ($\mathbf{I}'$), and **c**, the DOS probability matrix ($\mathbf{X}'$). In **b** and **c**, *M* and *N* are the horizontal and vertical grid sizes in a rectangular window, respectively. The black-filled entries indicate the nonzero values.

**4. Additional details on estimation of the coefficients of principal component vectors in binary systems**

For fitting coefficients ($\alpha'_p$) of the principal component (PC) vectors for a test binary alloy, we first need to calculate the feature ($n_d$, CN, and $F_{mix}$) values of the training data and test data. The values for the Cu-Fe systems considered in this work are summarized in Table S1. The coefficients ($\alpha_p$) of the PC vectors for the training systems should also be determined and can be automatically determined after the principal component analysis (PCA). Then, we generate linear regression lines between the $\alpha_k$ of the training data, in which we focus on the linear regression line between the two training data sets near the test composition. Then, based on the features of the training and test systems, we estimate each $\alpha'_k$ contributions of $n_d$, CN, and $F_{mix}$ $\{\alpha'^{n_d}_p, \alpha'^{\mathrm{CN}}_p, \alpha'^{\mathrm{F_{mix}}}_p\}$ for the test system by using the linear regression line. For example, in the case of the Cu-Fe system of this work, we considered five training systems: Cu, $Cu_{0.25}Fe_{0.75}$, $Cu_{0.5}Fe_{0.5}$, $Cu_{0.75}Fe_{0.25}$, and Fe. As a test system, we considered the $Cu_{0.375}Fe_{0.625}$ alloy. Based on the composition, the two training systems most similar to the test system are $Cu_{0.25}Fe_{0.75}$ and $Cu_{0.50}Fe_{0.50}$. Here, we note that the feature values for the training and test systems are summarized in Table S1. To estimate $\alpha'^{n_d}_{p=1,\mathrm{Cu_{0.375}Fe_{0.625}}}$, we consider the pink line ($p$ = 1) in Fig. S7 between the two training systems. In Table S1, the $n_{d,Fe}$ values of the training systems are 0.64 for $Cu_{0.25}Fe_{0.75}$ for and 0.38 for $Cu_{0.5}Fe_{0.5}$, the values of which are the boundary values of the pink line. A linear interpolation on the pink line with $n_{d,Fe}$ = 0.50 for the test $Cu_{0.375}Fe_{0.625}$ alloy provides the $\alpha'^{n_d}_{p=1,\mathrm{Cu_{0.375}Fe_{0.625}}}$ value. Using a similar approach, we can estimate $\alpha'^{\mathrm{CN}}_{p=1,\mathrm{Cu_{0.375}Fe_{0.625}}}$ and $\alpha'^{\mathrm{F_{mix}}}_{p=1,\mathrm{Cu_{0.375}Fe_{0.625}}}$. Then, $\alpha'_{p=1,\mathrm{Cu_{0.375}Fe_{0.625}}}$ is calculated by dividing the sum of the three $\alpha'_{p=1}$ values by 3. Other PC coefficients are estimated in a similar manner.

**Table S1. $n_{d,Cu}$, $n_{d,Ni}$, CN, and $F_{mix}$ of training and test data in the Cu-Fe binary system.** The asterisk indicates a test data, and the others are for training data.

| | $n_{d,Cu}$ | $n_{d,Fe}$ | CN | $F_{mix}$ |
|---|---|---|---|---|
| **Cu** | 1 | 0 | 12 | 0 |
| **$Cu_{0.75}Fe_{0.25}$** | 0.83 | 0.17 | 12 | 0.38 |
| **$Cu_{0.50}Fe_{0.50}$** | 0.62 | 0.38 | 12 | 0.51 |
| **$Cu_{0.25}Fe_{0.75}$** | 0.36 | 0.64 | 8 | 0.41 |
| **Fe** | 0 | 1 | 8 | 0 |
| **$*Cu_{0.375}Fe_{0.625}$** | 0.50 | 0.50 | 12 | 0.45 |

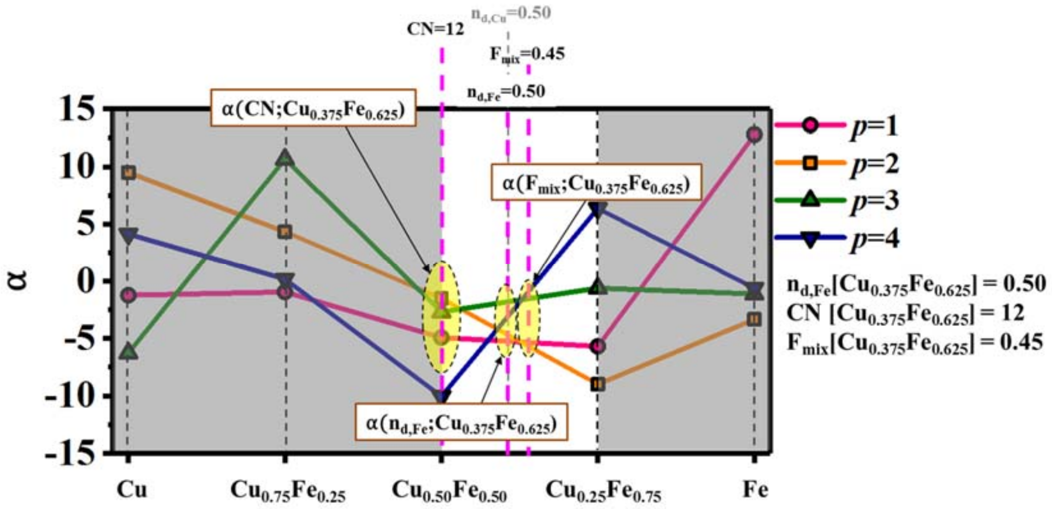


**Figure S7. Linear regression lines for estimation of coefficients of PC vectors using the feature values such as $n_d$, CN, and $F_{mix}$ in the Cu-Fe system.** *p* indicates the index of PC, where four PC components are considered. The coefficients of each PC for the test alloy are determined by the cross-points between the regression lines and the dashed lines corresponding to the feature values of the test alloy. The highlighted range is the estimated region of the test alloys.

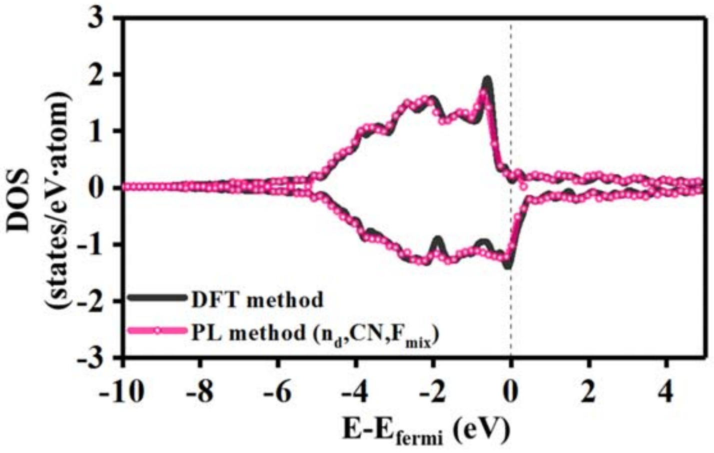


**Figure S8. DOS pattern of $Cu_{0.375}Ni_{0.625}$ as a test alloy.** The energy range ($E-E_{fermi}$) is from E = -10 eV to E = 5 eV, and the DOS range is from 0.0 to ±3.0 where the positive region is for up-spin and the negative is for down-spin. Black corresponds to the DFT method, and pink corresponds to the learning method using the three features ($n_d$, CN, and $F_{mix}$).

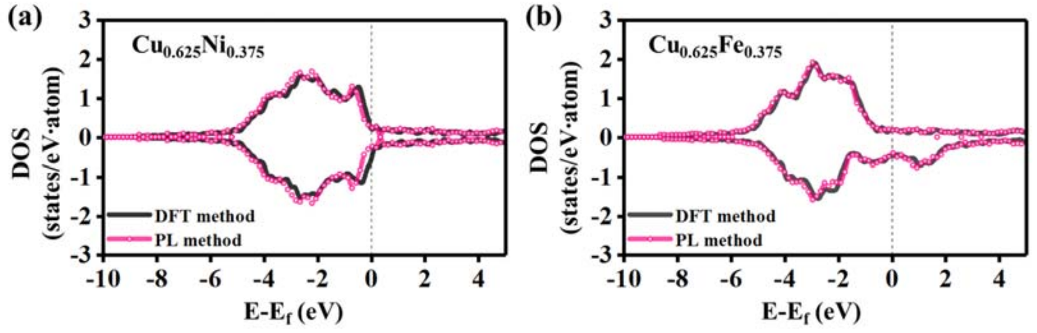


**Figure S9. DOS pattern of (a) $Cu_{0.625}Ni_{0.375}$ and (b) $Cu_{0.625}Fe_{0.375}$ using our pattern learning method.** The energy range ($E-E_{fermi}$) is from E = -10 eV to E = 5 eV, and the DOS range is from 0.0 to ±3.0 where the positive region is for up-spin and the negative is for down-spin. Black corresponds to the DFT method, and pink corresponds to the learning method using the three features ($n_d$, CN, and $F_{mix}$).

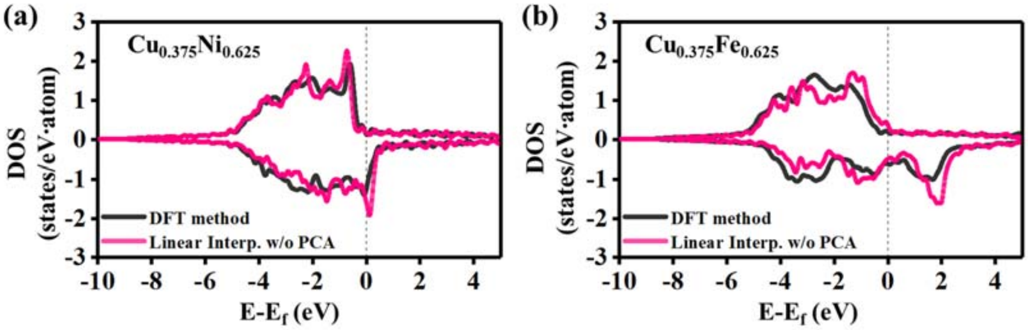


**Figure S10. DOS pattern of (a) $Cu_{0.375}Ni_{0.625}$ and (b) $Cu_{0.375}Fe_{0.625}$ by a linear interpolation of two nearest neighbors without PCA.** The energy range ($E-E_{fermi}$) is from E = -10 eV to E = 5 eV, and the DOS range is from 0.0 to ±3.0 where the positive region is for up-spin and the negative is for down-spin. Black corresponds to the DFT method, and pink corresponds to the linear interpolation of two nearest neighbors without PCA.

## 5. Estimation of the coefficients of PC vectors in a ternary system

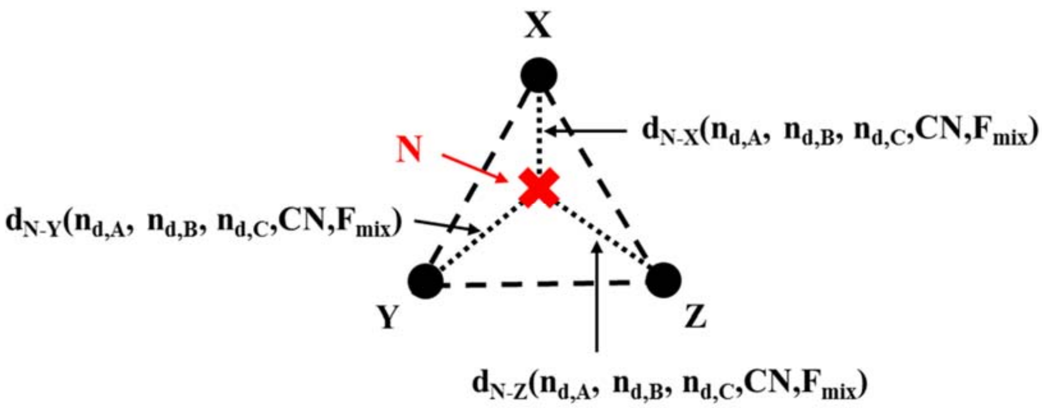


**Figure S11. Schematic triangular diagram in a ternary system to represent a distance between two compositions.** In the A-B-C ternary system, X, Y, and Z are training systems and N is the test system. $d_{N\text{-}X}$, $d_{N\text{-}Y}$, and $d_{N\text{-}Z}$ indicate the differences of each feature between N-X, N-Y, and N-Z.

**Table S2. $n_{d,Cu}$, $n_{d,Ni}$, $n_{d,Pt}$, CN, and $F_{mix}$ of training and test data in the Cu-Ni-Pt ternary alloy system.** The asterisk indicates a test data and the others are for training data.

| | $n_{d,Cu}$ | $n_{d,Ni}$ | $n_{d,Pt}$ | CN | $F_{mix}$ |
|---|---|---|---|---|---|
| **Cu** | 1.00 | 0.00 | 0.00 | 12 | 0.00 |
| **$Cu_{0.75}Ni_{0.25}$** | 0.79 | 0.21 | 0.00 | 12 | 0.41 |
| **$Cu_{0.75}Pt_{0.25}$** | 0.77 | 0.00 | 0.23 | 12 | 0.50 |
| **$Cu_{0.50}Ni_{0.50}$** | 0.56 | 0.44 | 0.00 | 12 | 0.50 |
| **$Cu_{0.50}Ni_{0.25}Pt_{0.25}$** | 0.54 | 0.22 | 0.24 | 12 | 0.65 |
| **$Cu_{0.50}Pt_{0.50}$** | 0.53 | 0.00 | 0.47 | 9 | 0.83 |
| **$Cu_{0.25}Ni_{0.75}$** | 0.29 | 0.71 | 0.00 | 12 | 0.35 |
| **$Cu_{0.25}Ni_{0.50}Pt_{0.25}$** | 0.29 | 0.46 | 0.26 | 12 | 0.63 |
| **$Cu_{0.25}Ni_{0.25}Pt_{0.50}$** | 0.28 | 0.22 | 0.50 | 12 | 0.68 |
| **$Cu_{0.25}Pt_{0.75}$** | 0.27 | 0.00 | 0.73 | 12 | 0.38 |
| **Ni** | 0.00 | 1.00 | 0.00 | 12 | 0.00 |
| **$Ni_{0.75}Pt_{0.25}$** | 0.00 | 0.73 | 0.27 | 12 | 0.50 |
| **$Ni_{0.50}Pt_{0.50}$** | 0.00 | 0.47 | 0.53 | 12 | 0.56 |
| **$Ni_{0.25}Pt_{0.75}$** | 0.00 | 0.23 | 0.77 | 12 | 0.39 |
| **Pt** | 0.00 | 0.00 | 1.00 | 12 | 0.00 |
| ***$Cu_{0.06}Ni_{0.06}Pt_{0.88}$** | 0.07 | 0.06 | 0.88 | 12 | 0.10 |
| ***$Cu_{0.31}Ni_{0.34}Pt_{0.34}$** | 0.35 | 0.31 | 0.34 | 12 | 0.62 |

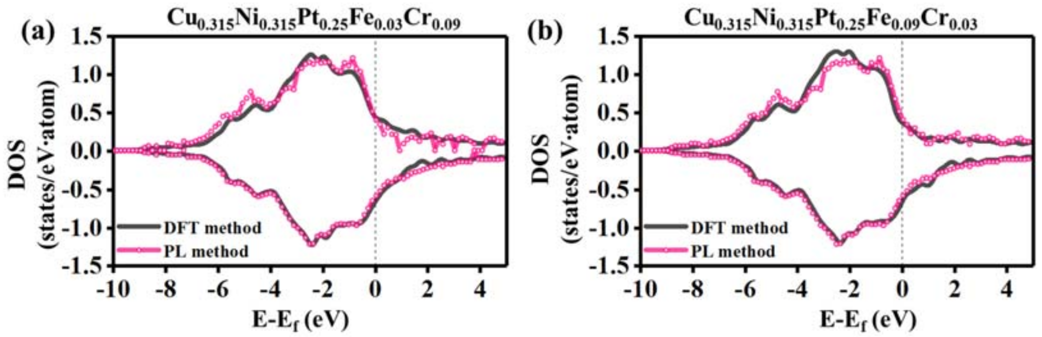


**Figure S12. DOS pattern of (a) $Cu_{0.315}Ni_{0.315}Pt_{0.25}Fe_{0.03}Cr_{0.09}$ and (b) $Cu_{0.315}Ni_{0.315}Pt_{0.25}Fe_{0.09}Cr_{0.03}$ as test compositions of high entropy alloys.** The energy range ($E-E_{fermi}$) is from E = -10 eV to E = 5 eV, and the DOS range is from 0.0 to ±1.5 where the positive region is for up-spin and the negative is for down-spin. Black corresponds to the DFT method, and pink corresponds to the learning method using the three features ($n_d$, CN, and $F_{mix}$).

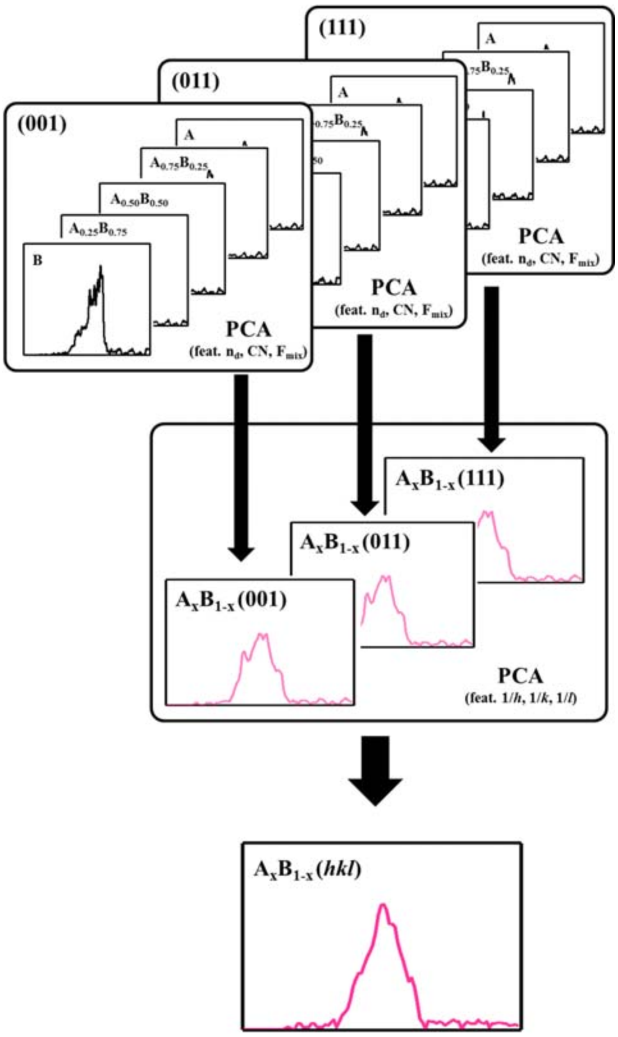


**Figure S13. Scheme of the pattern learning method for predicting the DOS pattern for a high-index surface of $A_xB_{1-x}$ alloys.** As a training system for the pattern learning method, five compositions (A, $A_{0.75}B_{0.25}$, $A_{0.50}B_{0.50}$, $A_{0.25}B_{0.75}$, and B) and three low index surfaces ((001), (011), and (111)) for each composition were considered. The process includes two steps. The *first* step is demonstrated in the upper and middle boxes shows the prediction of the DOS patterns for the low-index surface of the $A_xB_{1-x}$ alloy using the $n_d$, CN, and $F_{mix}$ features. The *second* step is demonstrated in the middle and the bottom boxes and shows prediction of the DOS pattern for the high-index surface (*hkl*) of the $A_xB_{1-x}$ alloy using the 1/*h*, 1/*k*, and 1/*l* features.

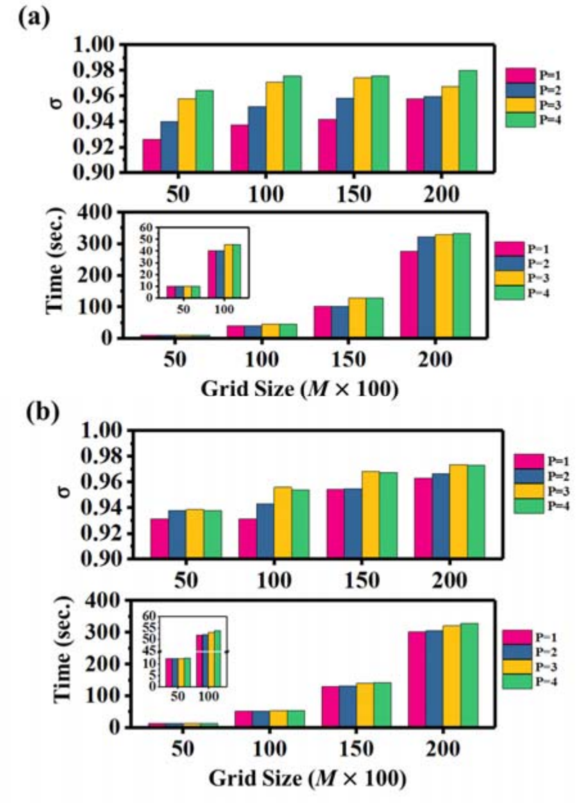


**Figure S14. Performance tests of (a) training structure ($Cu_{0.5}Ni_{0.5}$) and (b) test structure ($Cu_{0.375}Ni_{0.625}$) for the number of PCs and grid size in a Cu-Ni binary alloy system.** Relationships between the grid size and the pattern similarity of the learning model (upper side) and between the grid size and the calculation time (lower side). P indicates the number of PC eigenvectors used during the prediction process.
